# Clinical outcome of hypofractionated breath-hold image-guided SABR of primary lung tumors and lung metastases

**DOI:** 10.1186/1748-717X-9-10

**Published:** 2014-01-08

**Authors:** Judit Boda-Heggemann, Anian Frauenfeld, Christel Weiss, Anna Simeonova, Christian Neumaier, Kerstin Siebenlist, Ulrike Attenberger, Claus Peter Heußel, Frank Schneider, Frederik Wenz, Frank Lohr

**Affiliations:** 1 Department of Radiation Oncology, Universitätsmedizin Mannheim, Medical Faculty Mannheim, Heidelberg University, Mannheim, Germany; 2 Department of Biomathematics and Medical Statistics, Universitätsmedizin Mannheim, Medical Faculty Mannheim, Heidelberg University, Mannheim, Germany; 3 Institute of Diagnostic Radiology and Nuclear Medicine, Universitätsmedizin Mannheim, Medical Faculty Mannheim, Heidelberg University, Mannheim, Germany; 4 German Center for Lung Research, Diagnostic and Interventional Radiology with Nuclear Medicine, Thoraxklinik Heidelberg at Ruprecht-Karl-University, Heidelberg, Germany

**Keywords:** Hypofractionated intensity modulated breath-hold image-guided (ig)SABR, Lung tumors, Lung metastases, Local control, Survival, Toxicity

## Abstract

**Background:**

Stereotactic Ablative RadioTherapy (SABR) of lung tumors/metastases has been shown to be an effective treatment modality with low toxicity. Outcome and toxicity were retrospectively evaluated in a unique single-institution cohort treated with intensity-modulated image-guided breath-hold SABR (igSABR) without external immobilization. The dose–response relationship is analyzed based on Biologically Equivalent Dose (BED).

**Patients and methods:**

50 lesions in 43 patients with primary NSCLC (n = 27) or lung-metastases of various primaries (n = 16) were consecutively treated with igSABR with Active-Breathing-Coordinator (ABC®) and repeat-breath-hold cone-beam-CT. After an initial dose-finding/-escalation period, 5x12 Gy for peripheral lesions and single doses of 5 Gy to varying dose levels for central lesions were applied. Overall-survival (OS), progression-free-survival (PFS), progression pattern, local control (LC) and toxicity were analyzed.

**Results:**

The median BED2 was 83 Gy. 12 lesions were treated with a BED2 of <80 Gy, and 38 lesions with a BED2 of >80 Gy. Median follow-up was 15 months. Actuarial 1- and 2-year OS were 67% and 43%; respectively. Cause of death was non-disease-related in 27%. Actuarial 1- and 2-year PFS was 42% and 28%. Progression site was predominantly distant. Actuarial 1- and 2 year LC was 90% and 85%. LC showed a trend for a correlation to BED2 (p = 0.1167). Pneumonitis requiring conservative treatment occurred in 23%.

**Conclusion:**

Intensity-modulated breath-hold igSABR results in high LC-rates and low toxicity in this unfavorable patient cohort with inoperable lung tumors or metastases. A BED2 of <80 Gy was associated with reduced local control.

## Introduction

Standard therapy for stage I-II lung cancer or solitary lung metastases is surgical resection. SABR (Stereotactic Ablative Radiotherapy) is a non-invasive, effective and low-toxicity alternative for medically inoperable patients [1].

SABR of lung lesions poses a special challenge for several reasons such as the highly volume-dependent radiosensitivity of healthy lung tissue and surrounding organs at risk (OAR, e.g. oesophagus, heart), breathing-induced motion of pulmonary targets and the dosimetrically difficult situation of a high-density tumor lesion surrounded by low-density lung tissue. The lung itself is one of the most radiation-sensitive organs with two different manifestations of radiation damage with different time frames. As a severe early (subacute) side effect of radiation therapy, pneumonitis occurs in 5-15% 4–6 weeks after conventionally fractionated large-volume thoracic irradiation. The incidence of radiation pneumonitis depends on the radiation dose and the irradiated volume of the normal lung tissue [2]. As a late side effect and consequence of radiation pneumonitis, pulmonary fibrosis may arise, rendering the affected tissue without function.

In spite of potentially serious side effects, dose escalation is the most important part of improvement of local control of lung targets and therefore should be aimed at [3] since both model calculations [4] and clinical data suggest that doses necessary for tumor ablation are higher than initially thought [5]. To reliably and accurately create the required highly conformal radiation doses necessary for lung target irradiation, improvement of imaging, planning, dose calculation and delivery tools is needed.

The development of devices that enable image guided radiotherapy (IGRT) together with non-invasive lung immobilization makes a “frameless” stereotactic approach possible [6]. Breath-hold techniques for tumor immobilisation facilitate the delivery of high doses to the PTV while maximally sparing OAR [7]. Dosimetric comparisons between free-breathing and breath hold radiotherapy for lung cancer [8] have shown an improved target conformity index and less dose to the heart and healthy lung if compared to free breathing planning. Computer-controlled breath-hold with the ABC®-system (Active Breathing Coordinator, Elekta AB, Stockholm, Sweden) in combination with daily image-guidance has been successfully implemented for targets that move with spontaneous breathing [9].

Recent pooled analyses of almost 500 patients with stage I NSCLC have shown excellent results with local control rates of 92% and low toxicity [10]. Other large multicenter trials, for example the RTOG 1021 trial, comparing the sublobular resection with SABR in patient cohorts, with high risk of complications with more extensive surgery are currently in progress.

In this retrospective evaluation, we assess outcome and toxicity in a unique single-institution series of patients who received volume-image-guided, intensity modulated breath-hold lung SABR with both primary lung and metastatic lesions, undergoing no external immobilization.

## Patients and methods

### Patients

Between 2002 and 2009 50 lesions of 43 consecutive patients with NSCLC (n = 27, St. I-II in the primary situation and III-IV including patients with controlled brain metastases or local relapse after primary standard therapy) and lung metastases of various primary tumors (n = 16; 2 melanoma; 3 oropharyngeal, 1 laryngeal, 1 prostate, 4 colorectal, 1 pancreatic and 1 breast cancer; 1 transitional cell and 2 renal cell carcinoma) were consecutively treated with intensity-modulated breath-hold igSABR after informed consent. All lesions were considered to be technically or medically inoperable by an interdisciplinary tumor-conference. Patient and tumor characteristics are summarised in Table 1, while the NSCLC series is further described in detail in Table 2.

**Table 1:** Patients (n = 43) characteristics

**Characteristics**	**Total (Percentage)**
Gender:	
Male	32 (74%)
Female	11 (26%)
Age:	
Ys, Median; (range)	69 (49–84)
Tumor entities:	
NSCLC	27 (63%)
Lung metastasis of various primary tumors	16 (37%)

**Table 2:** Further characteristics of patients with NSCLC (n = 27)

**Characteristics**		**Staging**	
Histologic diagnosis Squamous cell carcinoma Adenocarcinoma others	9 (33%) 13 (48%) 5 (19%)	Stage(AJCC)	
		Ia	6 (22%)
		Ib	4 (15%)
		IIa	3 (11%)
		IIb	3 (11%)
		IIIa*	5 (19%)
		IIIb*	1 (3%)
		IV*	5 (19%)
Chemotherapy before or after RT		Tumor:	
		T1a	2 (7%)
yes	12 (45%)	T1b	10 (37%)
		T2a	5 (19%)
no	15 (55%)	T2b	1 (3%)
		T3	4 (15%)
		T4	5 (19%)
Comorbidity:		Lymphnodes	
		N0	18 (67%)
		N1	5 (19%)
Pulmonary disease (COPD)	6 (22%)		
Cardiovascular disease	7 (26%)		
Both	12 (45%)	N2	3 (11%)
None of the above	2 (7%)	N3	1 (3%)
		Metastasis:	
		M0	22 (82%)
		M1a	1 (3%)
		M1b	4 (15%)

Detailed characteristics of patients with NSCLC (n = 27).

*Patients treated with SBRT in relapse situation or with stable brain metastases based on patient wish and individual therapy decision.

Data were evaluated retrospectively regarding overall survival (OS), progression-free-survival (PFS), progression pattern, local control (LC), acute and late toxicity based on clinical symptoms and CTC/LENT-SOMA scales.

### Radiotherapy planning, dose calculation and treatment

Planning CT scans were acquired with a spiral-CT (Somatom Emotion, Siemens, Erlangen, Germany, thereafter Brilliance Big Bore Oncology, Philips, Hamburg, Germany) after an initial patient training session in inspiratory breath-hold at approximately 70% of vital capacity with ABC® [9]. Radiotherapy planning was initially performed as manually weighted Intensity Modulated RadioTherapy (IMRT) with OTP (Theranostic GmbH, Solingen, Germany) and thereafter with inverse planned step-and-shoot IMRT or VMAT (Volumetric Modulated Radiotherapy) with Monaco® (Elekta AB, Stockholm, Sweden).

PTV was calculated from CTV by adding a 5 mm margin radially and 10 mm in the craniocaudal direction to compensate residual intrafractional error of the ABC®-based positioning [11].

Dose calculation was performed initially by a pencil beam (PB) algorithm (11 patients), thereafter both with PB and collapsed cone (CC) algorithms (32 patients). After the change to CC algorithm, the PB was still calculated in order to compare the resulting nominal dose distributions.

Dose prescription was initially performed to the isocenter (forward-planned IMRT), typically in the vicinity of the median dose) and later as the median dose in the PTV (inverse IMRT) with the 90% isodose line covering the PTV. Dose constraints for OAR were as shown in Table 3[5,12-15]. The planning constraints are the constraints for the final dose level of typically 5x12 Gy. While we attempted to fulfill the planning goals whenever possible for this regimen, we had, however, for example for tumors close to the chest wall or the plexus, to deviate from these constraints on occasion depending on individual physician and patient preference if adhering to constraints would have precluded applying sufficient tumor dose. Dose constraints for the other regimens were adjusted for each regimen.

**Table 3:** **Dose constraints for OAR** (**OAR**, **Organs at risk**; **D**_**max**_, **maximal dose in PTV; V15 and V20, percentage of irradiated tissue covering the 15 or 20 Gy isodose [**[5,11-14]**])**

**OAR**	**Dose constraint**
Healthy lung V15	<30%
Healthy lung V20	<20%
Spinal cord D_max_	18-20 Gy
Trachea/main bronchus D_max_	36 Gy
Esophagus D_max_	18 Gy
Brachial plexus D_max_	18 Gy
Ribs/Thoracic wall D_max_	30 Gy
Heart and major vessels D_max_	30 Gy
Skin D_max_	40 Gy

PTV-coverage was analysed based on relevant parameters (D99 (dose encompassing 99% of the PTV), minimal, maximal, mean and median PTV-dose.

Implementing results from published literature reports [5,13-16] regarding dose escalation and fractionation, dose to the patients was adjusted during the reported period and varied between single-fraction doses of 20-26 Gy initially (depending on tumor and healthy lung volume) and various hypofractionated regimens with the current, final protocol prescribing 5x12 Gy every other day to peripheral tumors and 12x5 Gy to central lesions [15]. For exact fractionation schedules of each lesion, see Additional file 1: Table S1.

To be able to retrospectively compare these various fractionation regimens, we introduced Biologically Effective Dose in 2 Gy fractions (BED2 [17]). BED2 was calculated [18] with an assumed α/β ratio of 10 with the following formula: BED2 = Dx(d + α/β)/(2 + α/β).

Patients were treated as described previously [17]. Shortly, a linac with 6MV photons was used (Synergy®, Elekta AB, Stockholm, Sweden). Daily image-guidance was performed with EPIDs (Electronic Portal Imaging Device) and since 2005 with repeat breath-hold CBCT (XVI®, Elekta AB, Stockholm, Sweden [17,19]). Planning-CT images were matched online with the daily CBCT images using manual fusion with respect to soft-tissue anatomy [20]. Online surveillance of breath-hold was performed based on the continuous acquisition of MV-frames during irradiation allowing position verification of the tumor itself, if possible, or of a surrogate structure such as the diaphragm [6].

Patient follow-up (FU) was scheduled 6 weeks after radiotherapy and every 3 months thereafter with clinical examination and thoracic CT with i.v. contrast. An assessment of tumor response was performed using the RECIST (Response Evaluation and Criteria in Solid Tumors) criteria. Response was graded as complete response (CR), partial response (PR), stable disease (SD) or progression.

Acute (first 90days) and late toxicity (>90 days) was evaluated based on clinical symptoms (graded based on the CTC-scale in the acute phase and on the LENT-SOMA criteria (late effects in normal tissues subjective, objective, management and analytic scales) in the late phase). Recorded clinical symptoms were general condition, coughing, dyspnoea, pneumonitis, pulmonary oedema, dysphagia, pleural effusion, fever and skin symptoms for assessing acute toxicity; rib fracture, pulmonary fibrosis, thoracic pain, dyspnoea and coughing for late toxicity. Pneumonitis analysis was based on presence of symptoms requiring treatment and thoracic CT imaging.

### Statistics

Statistical analysis was performed with the SAS-software, release 9.01 (SAS, Cary, NC, USA). OS (Overall-Survival), PFS (Progression-Free-Survival) and LC (Local Control) were recorded and subject to actuarial analysis. OS was calculated from the day of irradiation until either the day of death (event) or the day of the last FU (censored data). PFS was calculated from the day of irradiation until either the day of relapse or death (events) or the last FU without relapse (censored data when at the last FU the patient lived without any evidence of progression). LC was calculated from the day of irradiation until either the day of local progression (event) or the last FU/death without local progression (censored data). For LC, number of patients at risk was calculated for each time point. Kaplan-Meier-plots for OS, PFS and LC were calculated in order to assess median survival/control times. Correlation of the local control time with PTV size and BED2 was analysed by the Kaplan-Meier log-rank test. P-values < 0.05 were considered as significant, 0.05 < p < 0.15 as a trend to significance.

## Results

### Radiotherapy data

All patients managed to achieve sufficient repeat breath-hold with ABC^®^.

Mean BED2 (Biologically Effective Dose) was 87±20 Gy (median 83 Gy). 12 lesions were treated with a BED2 of <80 Gy, and 38 lesions with a BED2 of >80 Gy (range 50-150 Gy). PTV-volume was 94±90 cm^3^ (median 69 cm^3^).

### Follow-up (FU), Overall-Survival (OS), Progression-free-survival (PFS) and Local Control (LC)

Median follow-up (FU) was 15 months for all patients. Median FU was 24 months for living patients. 12 patients are alive, 31 patients have died.

**Figure 1:**
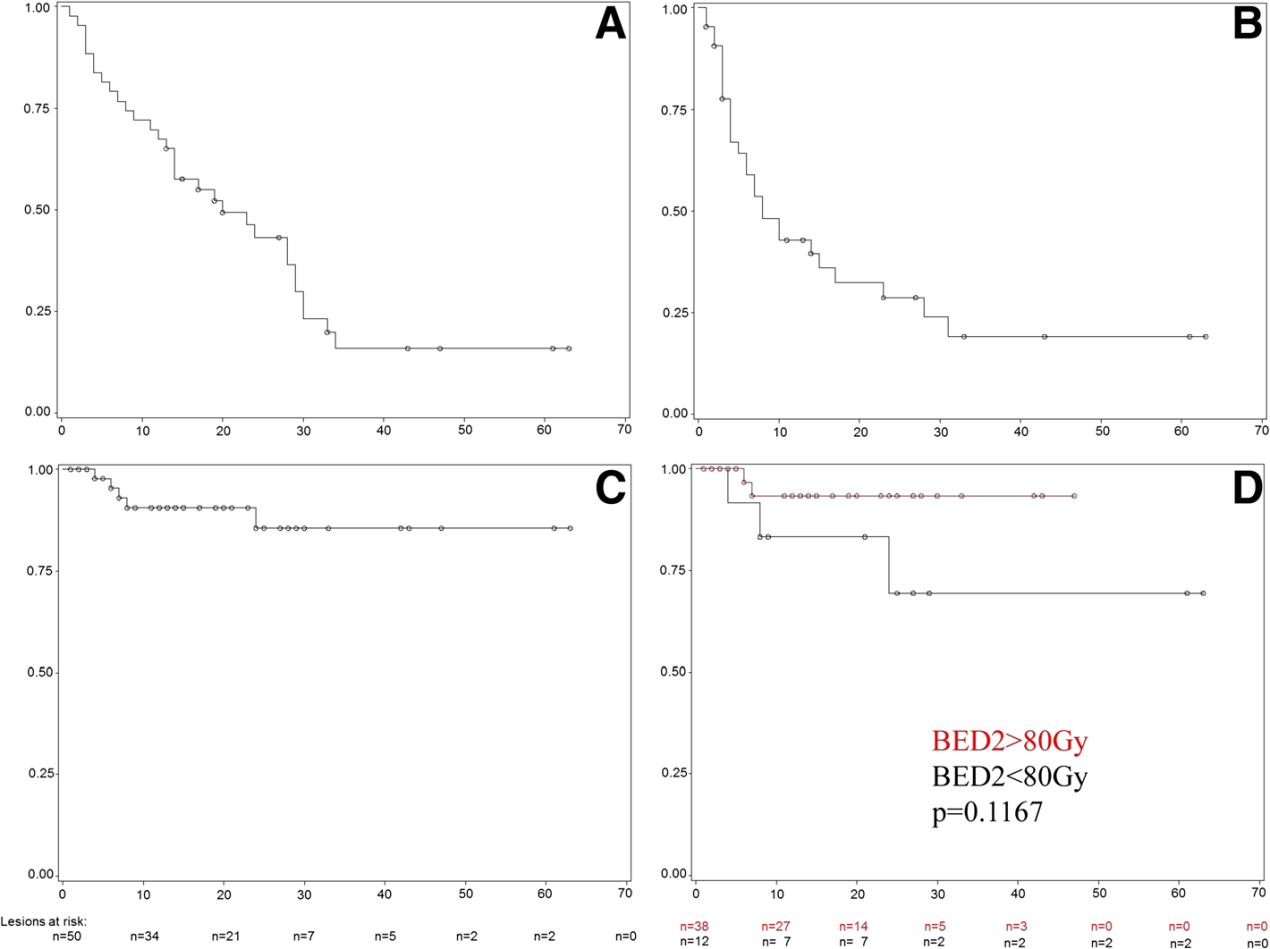
**Kaplan-Meier-plots for overall survival, progression free survival and local control. A**: Overall Survival of all patients, **B**: Progression Free Survival of all patients, **C**: Local Control of all lesions, **D**: Local Control for lesions treated with a BED2 > 80 Gy (red) and a BED2 < 80 Gy (black). The p-value shows a trend for a better local control with higher BED2. N: Number of lesions at risk at every given time-point.

Median OS was 20 months (95% CI: 13 – 29 months), without significant difference between patients with primary lung tumors or metastases (p = 0.3689). Actuarial 1-year OS was 67% (95% CI: 53% - 82%) and 2-year OS was 43% (95% CI: 27% - 59%) (Figure 1A). 28% of the patients died for non-disease related reasons (cardiovascular events or infections). Cause of death was systemic metastases outside the lung in 30% and progression of the primary tumor in 12% (1 oropharyngeal cancer, 4 primary lung tumor). Cause of death in one patient could not be determined with certainty although the date of death was confirmed by authorities. Actuarial 1-year PFS was 43% (95% CI: 27%-59%) and 2-year PFS was 29% (95% CI: 13%-44%; Figure 1B). Site of progression was predominantly distant (60% distant metastases (11 lung, 8 liver, 7 bone, 3 brain, 3 distant lymph node, 2 soft tissue, 2 spleen, 2 pleural, 1 peritoneal carcinomatosis, 1 adrenal gland, 1 kidney, 1 breast) and 32% mediastinal lymph nodes).

**Figure 2:**
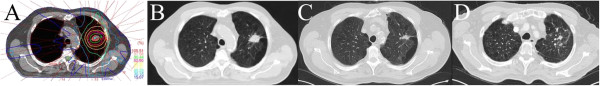
**Radiotherapy plan (A), pre-radiotherapy CT (B), follow-up CTs 2 months post-RT (C) and 1 year post-RT (D) of a typical patient.** Complete regression of the irradiated lesion can be observed without marked changes in healthy lung structure.

In 36 patients, size-reduction of the irradiated lesion was observed. In 5 patients, lesions remained unchanged; the lesion in one patient was progressive. Median time to response was 1.6 months (2.7 ± 2.6months (MV ± SD), typically at first reported FU). Complete response of the irradiated lesion was observed in 45 cases. Later local progression after initial regression was observed in 5 lesions after a median of 7 months. A typical patient example is shown in Figure 2.

Actuarial 1-year LC was 91% (34 patients at risk; CI: 82% - 99%) and 2-year LC was 86% (21 patients at risk; CI: 73% - 98%; Figure 1C). 95% of the lesions treated with a BED2 > 80 Gy were controlled locally after one year. No local relapses at doses >90 Gy BED2 were observed. Local progression was observed in only 5 cases, mainly in the initial dose finding phase, in one patient with an only retrospectively recognized pleural invasion of the irradiated lesion and in one patient with an extremely large PTV. Disregarding this case, the variation in PTV was not extensive (upper limit of tumor diameter: 5 cm) and no statistically significant influence of PTV size on local control was found in the range of diameters treated within this series. Several dose cut off values were tested statistically. Lesions with a BED2 > 80 Gy showed a trend for better local control (Figure 1D) than lesions treated with BED2 < 80 Gy. With the limited patient number, the difference is not statistically significant (p = 0.1167, Kaplan-Meier log rank). A comparison between NSCLC and patients with metastases did not show statistically significant difference in LC.

Most patients died due to systemic metastases with locally controlled irradiated lesions.

### Acute toxicity

7 patients had radiological signs of pneumonitis but no clinical symptoms (grade 1). Clinically apparent pneumonitis (requiring steroid treatment) was present in 23% (n = 9) of the treated patients. In six patients it was considered grade 2, while three patients developed a respiratory insufficiency as a consequence of their pneumonitis (grade 3). Two of them had suffered from severe chronic obstructive lung disease (COPD) before treatment and one of them already had a partial respiratory insufficiency. After conservative treatment of the grade 3 pneumonitis the functional condition of the patients was restituted to the pre-SABR state.

Given the fact that prior to SABR 19% (n = 8) of all patients had severe lung disease (mainly COPD), 36% (n = 15) had cardiovascular diseases (mainly coronary heart disease) and 31% (n = 13) suffered from both complicating diagnoses, which cause similar symptoms as radiation pneumonitis, we analyzed the changes in dyspnea and coughing from pre- to post-SABR with the CTC scale (Tables 4 and 5). No patient experienced fatal toxicity and no acute bleedings, fever or lung oedema were observed. Post-RT aggravation of dyspnea and coughing was observed in 8 of 9 clinically apparent pneumonitis cases. Grade 1 pleural effusions (with no therapeutic consequence) were observed in 7 cases.

**Table 4:** **Dyspnea and coughing as side effect in the acute and chronic phase after SABR**, **classified by CTC**

**CTC grade**	**Dyspnea**			**Coughing**		
	**Pre-treatment**	**Acute phase**	**Chronic phase**	**Pre- treatment**	**Acute phase**	**Chronic phase**
Grade 1	2	3	4	4	12	6
Grade 2	11	16	14	0	2	0
Grade 3	3	2	1	0	0	0
Grade 4	3	7	3	0	0	0

Patients, n = 43.

**Table 5:** Acute toxicity, reported separately for NSCLC (n = 27) and metastases (n = 16); dyspnea and coughing as grade difference between pre-therapeutic state and acute phase

**CTC grade**	**Δdyspnea**		**Δcoughing**		**Pneumonitis**	
	**NSCLC**	**met**	**NSCLC**	**Met**	**NSCLC**	**met**
Grade 1	2	3	2	3	5	2
Grade 2	2	2	1	0	2	4
Grade 3	0	0	0	0	1	2
grade 4	0	0	0	0	0	0

### Chronic toxicity

Late toxicity was difficult to analyse in this cohort of patients with advanced tumor stages due to progressive disease causing symptoms similar to radiation-induced changes. No rib fractures were observed. Radiation induced fibrosis LENT-SOMA grade 1 was observed in 12 patients in the follow-up CT, however, patients were clinically inapparent. After SABR, 6 patients developed chronic cough grade 1, with half of them already suffering from chronic bronchitis and COPD prior to SABR. In the patients alive at analysis, no change in dyspnea was registered in the chronic phase, when compared to that in the acute phase. However, evaluation is difficult (4 patients had died and 2 patients were lost to clinical FU).

## Discussion

SABR is a non-invasive therapeutic option for medically inoperable early stage lung cancer and lung metastases [21]. Dose escalation [5], which became possible due to improved planning and delivery techniques, has resulted in excellent local control rates, as detailed in Table 6: For stage I NSCLC, at 1 year after therapy, LC of 87-98% [1,15,21,29,36-43] and for advanced stage NSCLC or metastases 73-96% are achievable [5,16,22-28]. For pulmonary metastases, 2- and 3-yr OS rates of 47% and 32%, and 2- and 3-yr LC rates of 80% (both) with low toxicity were published recently [30].

**Table 6:** Literature overview SABR, mixed populations (tp = time point, * = only NSCLC stage I, -: not stated)

**Author year**	**N localisation**	BED2* (BED10)**	**OS (tp)**	**PFS (tp)**	**LC (tp)**
Wulf et al. [22]	51 lung/liver	50 Gy BED2	-	-	76% (1 y) 61-76% (2 y)
Hara et al. [23]	59 lung	50-125 Gy BED2	77% (1 y) 41% (2 y)	-	93% (1 y) 78% (2 y)
Yoon et al. [24]	101 lung	50-88 Gy BED2	51% (2 y)*	81% (2 y)	82% (2 y)
Milano et al. [25]	293 Oligometastases	31-72 Gy BED2	-	-	77% (2 y) 73% (4 y)
Norihisa et al. [26]	34 oligometastases	~75 Gy BED2	84% (2 y)	35% (2 y)	90% (2 y)
Salazar et al. [27]	109 NSCLC I-IV + oligometastases	120 Gy BED2	81% (2 y)* 48% (5 y)*	63%	82%
Rusthoven et al. [28]	63 lung	104-150 Gy BED2	39% (2 y)	37%	100% (1 y) 96% (2 y)
McCammon et al. [5]	246 lung/liver	150 Gy BED2	-	-	89% (>54 Gy, 3 yr)
Bradley et al. [12]	91 NSCLC I/II	71-126 Gy BED2	58% (3 y)	71% (3 y)	86% (2 y)
Duncker-Rohr et al. [29]	45 lung (NSCLC and metastases)	49.5-70.3 Gy BED2	52,7% (2 y)	-	80,5 (2 y)
Inoe et al. [30]	Lung metastases	106 Gy BED10 (30-168 Gy range)	47% (2 y) 32% (3 y)	40% (2 y) 32% (3 y)	80% (2 and 3 y)
Inoe et al. [31]	109 NSCLC I	66 Gy BED2	64% (5 y)	10% (5 y), intrathoracal progression	78% (5 y)
Verstegen et al. [32], SABR arm	64 NSCLC I-II	>100 Gy BED10	91.8% (1 y) 79.6% (3 y)	91.6% (1 y) 85.2% (3 y)	96.8% (1 y) 93.3% (3 y)
Kim et al. [33]	16 NSCLC I	88 Gy BED2 96 Gy BED2	87.5% (1.5 y)	85.2% (1.5 y)	91% (1.5 y)
Shioyama et al. [34]	8 SCLC stage I	88 Gy BED2	72% (3 y)	71% (3 y)	100% (3 y)
Grills et al. [35]	505 NSCLC I-IIB	132 Gy BED10	60% (2 y)	80% (2 y) 79% (3 y), distant metastases	94% (2 y)

*Biologically effective dose in 2Gy fractions (BED2Gy (for α/β 10) was calculated using the linear-quadratic model with an assumed α/β ratio of 10: BED2=Dx(d+α/β)/(2+α/β).

**BED(for α/β 10)=Dx(1+d/ α/β).

Despite our negatively selected patient cohort (high percentage of metastases of different primaries and 40% > Stage IIIA primary lung cancers), a 2-yr LC of 85% was achievable, which is comparable with the results in the literature in more favourably selected patients. LC in our cohort was independent of being a primary lung tumor or a metastasis, while available reports have suggested better results for (albeit typically earlier stage) primary lung tumors [44]. Regarding tumor-entities, our cohort was heterogeneous. This is, however, a situation similarly encountered in other SABR-series (e.g. [27,45]).

PFS at 2 years of 28% in our cohort is lower than published for early stage NSCLC (52-86%, [1,15,21,36-43]) or mixed cohorts (37-81%, [5,16,22-28]). This is probably due to the extremely negatively selected patients (all stages included) and high percentage of metastases. Progression occurred in mediastinal lymph nodes (12%; similar to literature reports [46]), or distantly (78%). Distant metastases indicate a clear need for detailed pretreatment staging (e.g. PET), appropriate selection criteria of patients for SABR alone and improving systemic therapy [16].

A 2-yr OS of 43% in our cohort is comparable with literature data (30-78% for NSCLC alone and 30-84% for mixed cohorts) with cause of death mainly being distant progress (42%), as in most series. This relatively long OS validates the LC data that might otherwise be biased by the chance of relapse being reduced by short survival.

This series suggests improved LC with higher doses, particularly if a BED2 > 80 Gy was applied, though this difference did not reach statistical significance in this relatively small cohort. LC after 1 year was maintained in >95% of the lesions which had been treated with a BED2 > 90 Gy. The reason for the nominally low “threshold” dose for durable local control may be found in the very high precision in dose delivery using igSABR, thus reducing the amount of “lost” dose. Similar results were reported in a recent manuscript by Duncker-Rohr et al. [29] with 2-yr LC of 95% (NSCLC) and 59,7% (metastases). Ablation doses might therefore not be as high as assumed at a time when treatment delivery was less spatially precise [47].

Toxicity was low despite many comparatively large lesions in the series. In RTOG-0236, grade 3 and 4 pulmonal side effects were recorded. In our cohort, no worsening of post-therapeutic symptoms > grade 3 (if compared to pre-therapeutic symptoms) was recorded. We did not observe any rib fractures [13,41] or skin toxicity > grade 2. RTOG 0915 will provide further insight as to what fractionation regimen to use, comparing different fractionation patterns regarding grade 3 toxicities.

Normal lung tolerance forbids, however, the application of *very* high doses for *centrally* located or *very* large tumors (>5 cm); which results in more frequent local relapses in such larger GTVs [21]. The results of RTOG-0813 should provide us with a recommendation for an effective dose that can be applied to Stage I central lesions with acceptable toxicity. Additional dose escalation in the future may be possible by further PTV-margin reduction through improvement and clinical integration of immobilization/tracking methods (breath hold, gating, online tracking [48]). If currently prohibitive technical limitations in beam application are overcome and immobilization and imaging methods known from photon therapy can be implemented successfully, particle therapy might further improve efficacy [49-53].

Regarding the role of adjuvant chemotherapy in addition to SABR, CALGB and RTOG are currently preparing respective trials [54]. In addition, antibodies, biologicals and radiosensitisers are also under investigation [55,56].

## Conclusions

Intensity modulated, image-guided breath-hold SABR is an effective non-invasive treatment modality that enables the application of reasonably high BED2 which in turn results in a high local control rate and relatively low toxicity in this negatively selected cohort of patients with inoperable lung tumors and lung metastases. Doses for tumor ablation may be lower than assumed at a time when delivery techniques were less precise. As disease progression was mainly outside the treated area, systemic therapy has to be further optimized in conjunction with SABR.

## Competing interest

This work was in part supported by a grant from Elekta AB, Stockholm, Sweden.

## Authors’ contributions

JBH drafted the manuscript and participated in the design and coordination of the study. AF collected the patient- as well as the radiation data and contributed equally with JBH in drafting the manuscript. CW performed the statistical analysis. AS, CN and KS assisted with the collection of radiation data. UA supported the interpretation of pre- and post-SBRT imaging. CPH allocated additional post-treatment CT images. FS provided support within the area of radiation oncology physics. FW and FL conceived of the study concept, participated in all aspects of its design and coordination and helped to draft the manuscript. All authors read and approved the final manuscript.

## Supplementary Material

Additional file 1: Table S1 F=fractions, D=daily dose, other= 3x20 Gy, 5x7 Gy and 2 times 11x5 Gy and 10x5 Gy, respectively. Click here for file
