# Supplementary material for: Clinical outcome of hypofractionated breath-hold image-guided SABR of primary lung tumors and lung metastases
Source: Radiat Oncol. 2014 Jan 8;9:10. doi: 10.1186/1748-717X-9-10 (PMC3909294; doi:10.1186/1748-717X-9-10)
Supplement: Additional file 1: Table S1 — F=fractions, D=daily dose, other= 3x20 Gy, 5x7 Gy and 2 times 11x5 Gy and 10x5 Gy, respectively. [file 1748-717X-9-10-S1.docx]

*Supplementary tables*

| Fractionation schedules (FxD) | Amount (n=50) |
| --- | --- |
| 5x10Gy | 16 (32%) |
| 5x11Gy | 12 (24%) |
| 5x12Gy | 9 (18%) |
| 5x8Gy | 3 (6%) |
| Single dose (range: 20-26Gy) | 4 (8%) |
| Other | 6 (12%) |

F=fractions, D=daily dose, other= 3x20Gy, 5x7Gy and 2 times 11x5Gy and 10x5Gy, respectively

| Delivered BED2 (range) | Amount (n=50) |
| --- | --- |
| 40-60 Gy | 6 (12%) |
| 60-80 Gy | 6 (12%) |
| 80-100 Gy | 28 (56%) |
| 100-120 Gy | 9 (18%) |
| 120-140 Gy | 0 |
| 140-160 Gy | 1 (2%) |
